# Prenatal exposure to extreme ambient heat may amplify the adverse impact of Superstorm Sandy on basal ganglia volume among school-aged children

**DOI:** 10.1371/journal.pone.0324150

**Published:** 2025-06-11

**Authors:** Donato DeIngeniis, Melissa Blum, Rebecca M. Lee, Ahmed Duke Shereen, Yoko Nomura

**Affiliations:** 1 Department of Psychology, Queens College, City University of New York, Queens, New York, United States of America; 2 Department of Psychology, The Graduate Center, City University of New York, New York, New York, United States of America; 3 Department of Environmental Medicine and Public Health, Icahn School of Medicine at Mount Sinai, New York, New York, United States of America; 4 Advaned Science Research Center at The Graduate Center, Neuroscience Initiative, City University of New York, New York, New York, United States of America; 5 Department of Psychiatry, Icahn School of Medicine at Mount Sinai, New York, New York, United States of America; Massachusetts General Hospital, UNITED STATES OF AMERICA

## Abstract

**Background:**

Weather-related stressors on healthy brain development has become an important topic in recent years. Notably, prenatal stress exposure to natural disasters may disrupt child neurodevelopment, with recent research exploring its impact on child brain morphology. Prenatal exposure to extreme weather events, such as ambient heat, may also affect child brain morphology. The basal ganglia, while historically related to motor ability, has gained increasing attention for its role in various non-motor functions, such as emotion regulation. Leveraging an existing cohort with and without prenatal exposure to Superstorm Sandy (SS), a category 3 hurricane at its peak, this study aims to investigate how prenatal exposure to both a natural disaster and extreme ambient heat impacts this important subcortical region.

**Methods:**

Main effects of SS and extreme heat exposure on basal ganglia volume were first analyzed to examine the independent effect on brain outcomes. Moderation models subsequently explored the potential role extreme heat had on the association between SS and basal ganglia volume. We used magnetic resonance imaging to measure basal ganglia gray matter volume at age 8 among 11 SS-exposed and 23 non-exposed children.

**Results:**

The SS-exposed group, relative to their non-exposed counterparts, had significantly larger volume in the putamen and pallidum bilaterally, and right caudate. No notable main effects of extreme heat were found. Moderation models revealed, however, extreme heat exposure amplified the adverse impact of SS exposure on basal ganglia volume, evidenced by reduced left nucleus accumbens and increased left pallidum volume.

**Conclusions:**

Prenatal exposure to SS impacted child brain development. Extreme heat amplified this risk via increased and reduced brain volume from different basal ganglia subregions. Alongside promoting initiatives to combat climate change, increasing awareness of the potential dangers of exposure to extreme climate events for pregnant individuals is vital for protecting long-term child brain development.

## Introduction

Climate change is increasing ambient temperature and causing natural disasters (e.g., hurricanes, tropical storms, wildfires, and droughts) with heightened frequency and severity. In 2012, Superstorm Sandy (SS), a category 3 hurricane at its peak in the Caribbean, made landfall in the New York City Metro area as a post-tropical cyclone. The storm caused extensive damage and ranked as one of the deadliest and most destructive storms in U.S. history [1]. Climate evidence suggests several warming-related factors (e.g., above-average sea surface temperatures, sea ice loss in the Arctic, and increased atmospheric moisture) may have intensified the storm’s impact [2]. Moreover, almost all the warmest years on record have occurred since 2000 and climate models project the next five years will be the hottest 5-year period on record [3,4].

While exposure to natural disasters and extreme ambient heat poses physical and mental health risks to the general population, certain groups, including pregnant individuals, especially those living in urban and under-resourced areas, are more vulnerable to its effects [5,6]. Pregnancy induces various physiological changes to the body, which makes thermoregulation more challenging and increases the risk of hyperthermia [7]. It is crucial to recognize that the negative consequences of exposure to prenatal stress are not limited to pregnant individuals. Exposure to stress during pregnancy increases the risk of poorer neurodevelopmental outcomes among child offspring [8,9]. This may initially present as disrupted emotion regulation mechanisms, such as difficulty with managing emotions and coping with stressful postnatal experiences [10]. As children grow, these early disruptions to emotion regulation may increase vulnerability to neurodevelopmental challenges and alterations in structural brain volume and functional activity. These effects are shaped by multiple factors, including the timing of exposure, genetic predispositions, and postnatal experiences [10].

Numerous studies have found associations between non-climate related stressors, such as maternal anxiety and/or depression, and greater externalizing (e.g., hyperactive, and aggressive) and internalizing (e.g., anxious and depressive) behavioral symptoms among child and adolescent offspring [11–16]. These associations between perceived stress and child behavioral symptoms have been replicated with measurable climate-related stressors, such as exposure to natural disasters (e.g., the 1998 Quebec Ice Storm and SS), via quasi-experiments among human populations [17,18].

As introduced above, exposure to extreme heat significantly threatens human physical health. It is important to recognize that various sociodemographic, infrastructural, and environmental factors strongly influence an individual’s susceptibility to adverse consequences from heat. For example, evidence suggests that those who represent a lower socioeconomic status (SES) and/or minority race are far more likely to experience negative consequences from heat exposure [19]. Moreover, heat morbidity is worsened in urban areas, like New York City, due to the urban “heat island” where man-made surfaces (e.g., concrete and dark roofs) absorb most of the sunlight and re-radiate it as heat while less vegetation is present to cool the air by evapotranspiration [20]. While studies examining the impacts of prenatal exposure to extreme heat on child mental health outcomes are still in their early stages, extreme heat exposure *in-utero* has been strongly linked to adverse obstetric outcomes (e.g., preterm birth and low birthweight) via reduced blood flow to the placenta, dehydration, and increased inflammation [7,21]. Of note, adverse obstetric outcomes among pregnant parents are a risk factor for various suboptimal developmental outcomes in children (e.g., impaired cognition, reduced physical growth, chronic health issues, and neuropsychiatric disorders) [22].

Although the link between climate and non-climate-related prenatal stress and increased child problem behaviors has gained considerable support, less attention has been paid toward understanding their consequences for child brain development. Optimal fetal brain development is critical as the brain grows most rapidly during the fetal period, reaching more than a third of the volume of the adult brain by the time of birth [23]. Neural phenotyping using magnetic resonance imaging (MRI) has been employed to help understand the biophysiological impacts of prenatal stress on child brain development. Non-climate-related (e.g., prenatal anxiety, depression, and normative stress) and climate-related (e.g., Quebec Ice Storm and Superstorm Sandy) stressors have been associated with discernible alterations in the gray matter volume and thickness of various cortical (e.g., prefrontal cortex) and subcortical (e.g., amygdala) brain regions among child offspring [24–29]. However, these findings remain limited and elusive, as studies contain mixed results with both smaller and larger brain volumes being reported.

The basal ganglia, a group of subcortical nuclei comprised of the striatum (caudate-putamen), pallidum (globus pallidus and ventral pallidum), and nucleus accumbens (NAcc) [30,31], is a promising region for examining optimal child neurobehavioral development. While initially thought to facilitate voluntary motor regulation and functioning [31,32], it has gained increasing attention in recent years for its ability to influence various non-motor functions, including executive functioning, working memory, language, reward valuation, and emotion recognition [32,33]. The non-motor role of the basal ganglia is evidenced by neuroimaging studies which have found basal ganglia subregions to form extensive circuits with limbic structures during affective tasks [32,33]. Moreover, functional alterations in the basal ganglia have been detected in various non-motor-related psychiatric disorders, such as anxiety, depression, and obsessive-compulsive disorder (OCD) [34].

Preliminary evidence has found strong associations between non-climate-related prenatal stress and structural alterations in basal ganglia brain volume. Prenatal mental health problems have been associated with decreased volumes in the basal ganglia, particularly the putamen among child offspring [35]. In contrast, increased volumes in the caudate, putamen, and pallidum during infancy have been attributed to maternal antenatal depression [36,37]. Our own recent work using a machine learning algorithm on a smaller sample of the stress in pregnancy (SIP) cohort found *in-utero* SS exposure to be highly associated with a larger right caudate [29]. It is important to conduct additional research to clarify these associations as they remain varied with both smaller and larger brain volumes reported.

Despite its potential importance, no study to date has investigated the individual and joint impact of two prominent and interconnected climate-related prenatal stressors, such as SS and ambient heat, on the brain volume of the basal ganglia among school-aged children. The study investigates the independent associations of both SS and extreme heat (main effects) and the interaction of the two (joint effect) on the brain volume of the core regions of the basal ganglia (i.e., caudate, putamen, pallidum, and nucleus accumbens). First, we investigate whether exposure to SS and extreme heat will individually lead to discernible differences in basal ganglia structures. We next explore whether exposure to extreme heat *in-utero* will amplify the risk prenatal exposure to SS poses on basal ganglia brain development as measured by changes in brain volume.

## Methods

### Study population

Participants were pooled from the SIP Study, a longitudinal project that follows parent/child dyads to understand the long-term impact of stress exposures during pregnancy. Pregnant individuals were recruited from large community-serving antenatal OB/GYN clinics at Mount Sinai Hospital and New York-Presbyterian/Queens in New York City during their second trimester. For this imaging pilot study, recruitment began on July 13, 2019, and ended on December 21, 2022. Thirty-four (*n* = 34) school-aged children at approximately age 8 (mean = 8.70, SD = 1.94) participated in the study. Of those children, 11 (9 females) were and 23 (15 females) were not exposed to SS *in-utero*. Seven (5 females) of the 11 SS exposed and 17 (10 females) of the SS unexposed children were exposed to extreme heat. Written informed consent was obtained from parents or guardians on behalf of their children, and the protocol received Institutional Review Board approval (Approval Number: 2018–1305-QC) at the City University of New York (CUNY). Inclusion criteria included being pregnant during the recruitment phase with intent of delivery. Exclusion criteria for participation in the parent study included HIV infection, maternal psychosis, maternal age < 15 years, life-threatening maternal medical complications, and congenital or chromosomal abnormalities in the fetus. Full details can be found in our cohort profile paper [38]. Additional exclusion criteria for MRI participation included metal implants, devices, and/or objects in the body.

### Measures

#### Superstorm Sandy (SS) exposure.

Children were classified as SS exposed if their parent was pregnant when SS made landfall. Children were SS unexposed if their parent was pregnant either before (Jan. 2011 – Oct. 2012) or after (Sept. 2013 – Oct. 2014) the storm made landfall.

#### Extreme heat exposure.

Participants were classified as extreme heat exposed if they were exposed to at least one day with a high temperature greater than 95 degrees Fahrenheit - 95°F (35°C) during any trimester of their pregnancy.

We extracted daily temperature estimates from three weather stations in New York City (Central Park, LaGuardia Airport, and John F. Kennedy Airport). Using the participants estimated delivery date (EDD) and gestational age at birth, we determined if the participants were exposed to days over 95°F (35°C) during their pregnancy based on the weather station closest to the longitude and latitude coordinates of their home address. EDD was estimated by sonogram at the first prenatal visit or self-reported last menstrual period (LMP). The temperature data was pulled from weather stations ranging from 7.50 to 23.27 kilometers (4.66 to 14.46 miles) apart. Additionally, the distance between participants’ residential addresses and their corresponding weather stations ranged from 0.87 kilometers (0.54 miles, nearest) to 12.21 kilometers (7.59 miles, farthest).

#### MRI assessment.

Participants were scanned with a Siemens 3 Tesla MRI scanner at the CUNY Advanced Science Research Center using a 32-channel head radio frequency receive coil. High resolution structural T1-weighted magnetization prepared rapid gradient echo (MPRAGE) data were acquired in the sagittal plane (inversion time/repetition time/echo time = 1070/2500/2.9 ms, flip angle = 8.0 degrees, field of view = 256 mm, matrix = 256 × 256, slices = 176, resolution = 1mm isotropic). Real-time motion detection and correction were implemented using Volumetric Navigators (vNav) [39], and the participants were asked to lie as still as possible.

The FreeSurfer pipeline (version 7.4.1) was used to generate cortical and subcortical volumetric measures [40]. The skull was stripped from the T1 images and the interface between the white and gray matter was estimated, which was further refined to obtain the thickness of gray matter. Cortical surfaces were inflated and Talairach transformation was performed. The cortex was parcellated into different anatomical regions using the Destrieux atlas [41]. Regional brain volumes were normalized by the total intracranial volume and were multiplied by 100 for percentage reporting. Analyses focused on the bilateral caudate, putamen, pallidum, and nucleus accumbens.

#### Potential confounders.

Child age at MRI assessment, biological sex, objective challenge related to SS, family socioeconomic status (SES), normative prenatal stress, age of the pregnant individual at the time of their child’s birth, race, the number of days exposed to the threshold temperature (i.e., > 95°F(35°C), season of conception, and the New York City Heat Vulnerability Index (HVI), were considered *a priori* as potential confounders and were included in the relevant main effect and interaction models for statistical adjustment.

Objective challenge related to SS was assessed by the Storm32 scale [42], which measures important aspects of disaster exposure within 30 days after the disaster. Examples include: as a result of Superstorm Sandy, did your residence suffer damage; did your family stay together for the duration of the storm; did you experience lack of potable water? Normative prenatal stress was extracted using latent profile analysis [43] with pregnancy-related anxiety, depression symptoms, anxiety symptoms, perceived stress, and stressful life events. Family SES index was extracted using latent class analysis with maternal education, pre-pregnancy occupation prestige, and work and welfare status (see details in [18]). Season of conception was determined based on the dates of the equinox (spring and autumn) or solstice (winter and summer) in the year the pregnant person conceived. The Heat Vulnerability Index (HVI) identifies neighborhoods in New York City whose residents face a higher risk of mortality following extreme heat exposure [44]. Neighborhoods are scored from 1 (lowest risk) to 5 (highest risk). The factors represented in the HVI include daytime surface temperature, access to green space and home air conditioning, and the percentage of residents who identify as low-income or non-Hispanic Black [44].

### Statistical approach

Normality tests on basal ganglia brain outcomes associated with SS and extreme heat were conducted using visual (Q-Q plot) and statistical (Shapiro-Wilk) methods. Appropriate normalization was applied if the assumption of normality was violated. Multicollinearity was addressed by testing the variance inflation factor (VIF) for all main effect and interaction models. Multicollinearity was not considered a threat if the VIF remained below 10 [45].

A series of linear regressions via general estimating equations (GEE) were conducted using IBM SPSS Statistics Version 29 (IBM Corporation, Armonk, NY) to determine potential group differences in basal ganglia gray matter volumes. Main effect models tested group differences by exposure to SS (yes/no) and extreme heat (yes/no) individually, and interaction models tested the impact of joint exposure via the potential moderation of extreme heat on the association between *in-utero* SS exposure and brain structure during middle childhood. Regressions were conducted with GEE to account for potential within-subject correlation of responses (i.e., intrafamilial correlations) [46,47]. For all analyses, potential confounders and intrafamilial correlations (i.e., four siblings) were adjusted. Models with significant associations (*p* < .05) were corrected for multiple testing via the Benjamini-Hochberg procedure with a 15% false discovery rate (FDR). FDR corrected *p*-values (adj-*p*) at <.15 were considered statistically significant. Significant main effect models report the percent volume difference in the mean basal ganglia gray matter volumes between the unexposed and exposed groups.

## Results

### Regression diagnostics

Apart from the left (*W* = .93, *p* = .03) and right (*W* = .93, *p* = .03) putamen, none of the brain outcomes potentially associated with SS and extreme heat violated normality (*p*’s > .05). Log10 transformations were applied to the putamen volumes to address the apparent positive skew in the data. Multicollinearity was not deemed a significant threat for the main effect and interaction models tested.

### Demographics

Except for child age at MRI assessment, no demographic differences were detected between the SS exposed and unexposed children. The age difference occurred due to more children being born prior to the storm than after. Full demographic differences are summarized in Table 1.

**Table 1:** Demographic and Environmental Characteristics of Participants by Superstorm Sandy Exposure Status.

Superstorm Sandy Status				
	Total (n = 34)	Exposed (n = 11)	Unexposed (n = 23)	
**Demographic & Environmental Variables**	Mean (SD)			Statistics, *p*-value
**Child Age at MRI Assessment**	8.70(1.94)	7.21(1.55)	9.41(1.71)	*F*(1,32) = 13.01, ***p*** **= .001**
**Maternal Age** ^a^	25.82(6.18)	27.09(6.66)	25.21(6.00)	*F*(1,32) = 0.68, *p* = .42
**Objective hardship** ^b^	2.58(2.55)	3.10(3.41)	2.33(2.06)	*F*(1,32) = 0.68, *p* = .42
**No. of Days >95°F**	3.18(2.67)	3.91(3.39)	2.83(2.25)	*F*(1,32) = 1.24, *p* = .28
N (%)				
**Child Sex Assigned at Birth**				*X*^2^(1) = 0.99, *p* = .32
Male	10(29.4)	2(18.2)	8(34.8)	
Female	24(70.6)	9(81.8)	15(65.2)	
**Maternal Race**				*X*^2^(3) = 1.73, *p* = .63
White	11(32.4)	4(36.4)	7(30.4)	
Black	10(29.4)	2(18.2)	8(34.8)	
Asian	1(2.9)	0(0.0)	1(4.3)	
Multicultural	12(35.3)	5(45.5)	7(30.4)	
**Prenatal Stress**				*X*^2^(2) = 0.01, *p* = .99
Low	12(35.3)	4(36.4)	8(34.8)	
Medium	19(55.9)	6(54.5)	13(56.5)	
High	3(8.8)	1(9.1)	2(8.7)	
^*^ **New York City HVI** ^c^				*X*^2^(1) = 1.79, *p* = .18
Low	16(47.1)	7(63.6)	9(39.1)	
High	18(52.9)	4(36.4)	14(60.9)	
**Family SES** ^d^				*X*^2^(2) = 1.40, *p* = .50
Low	2(5.9)	1(9.1)	1(4.3)	
Medium	18(52.9)	7(63.6)	11(47.8)	
High	14(41.2)	3(27.3)	11(47.8)	
**Season of Conception**				*X*^2^(3) = 3.30, *p* = .35
Winter	3(8.8)	2(18.2)	1(4.3)	
Spring	8(23.5)	1(9.1)	7(30.4)	
Summer	10(29.4)	3(27.3)	7(30.4)	
Autumn	13(38.2)	5(45.5)	8(34.8)	

**NB:**

^a^assessed at the time of childbirth;

^b^Objective Challenges related to Superstorm Sandy within 3 months;

^c^HVI = Heat Vulnerability Index;

^d^SES = socioeconomic status.

* Low HVI group = HVI score of 1–3 and High HVI group = HVI score of 4–5.

### Individual effect of Superstorm Sandy (SS) and extreme heat exposure on child brain volume

Main effects of *in-utero* SS and extreme heat exposure on child brain volume at age 8 were examined. *In-utero* SS exposure was associated with a larger left (+6.35%, adj-*p* = .001) and right (+6.22%, adj-*p* < .001) putamen, larger left (+1.89%, adj-*p* = .04) and right (+1.71%, adj-*p* = .007) pallidum, and a larger right caudate (+3.92%, adj-*p* = .08). Apart from the marginal association between extreme heat and a larger left pallidum (*p* = .09), no associations were found with extreme heat exposure. Full numerical results are summarized in Table 2 (SS) and Table 3 (heat). Supplementary figures S1 Fig (SS) and S2 Fig (heat) provide visual representations of these main effects.

**Table 2:** SS Exposure on Basal Ganglia Structural Volumes.

Brain region	Left hemisphere mean (SE) (n = 34)		Right hemisphere mean (SE) (n = 34)		Main Effect Statistics					
	Superstorm Sandy Exposure Status				Left hemisphere			Right hemisphere		
	Not Exposed	Exposed	Not Exposed	Exposed	Wald X^2^	*p*	adj *p*	Wald X^2^	*p*	adj *p*
Caudate vol.	32.10 (0.76)	33.90 (1.47)	32.53 (0.79)	36.45 (1.60)	1.03	.31	.35	3.92	.05	**.08**
Putamen vol.	44.02 (0.01)	50.37 (0.01)	44.70 (0.01)	50.92 (0.01)	12.92	<.001	**.001**	18.20	<.001	**<.001**
Pallidum vol.	16.64 (0.39)	18.53 (0.58)	15.68 (0.29)	17.39 (0.49)	5.55	.02	**.04**	9.15	.002	**.007**
NAcc vol.	5.10 (0.15)	5.34 (0.29)	5.31 (0.15)	5.94 (0.32)	.42	.52	.52	2.46	.12	.16

**NB:** the model includes sex and age of the child at MRI assessment, age and race of the pregnant person, prenatal stress class, and objective challenges related to SS and socioeconomic status (SES) class as covariates. Intrafamilial correlations (n = 4 siblings) were adjusted for. Brain volumes are normalized by total intracranial volume. Analyses were based on GEE. Standard errors for the left and right putamen reflect log10 transformations. FDR corrected p-values (adj *p*) were conducted with the Benjamini-Hochberg procedure with an FDR of 15%. NAcc = nucleus accumbens; vol = volume; SE = standard error. Values represent mean volume (%) and standard error*.*

**Table 3:** Extreme Heat Exposure on Basal Ganglia Structural Volumes.

Brain region	Left hemisphere mean (SE) (n = 34)		Right hemisphere mean (SE) (n = 34)		Main Effect Statistics			
	Extreme Heat (>95 Degrees) Exposure Status				Left Hemisphere		Right hemisphere	
	Not Exposed	Exposed	Not Exposed	Exposed	Wald X^2^	*p*	Wald X^2^	*p*
Caudate vol.	32.15 (2.04)	32.90 (0.79)	33.79 (2.25)	33.80 (0.81)	.09	.76	.000	.99
Putamen vol.	43.48 (0.02)	46.73 (0.01)	43.75 (0.02)	47.57 (0.01)	1.14	.29	1.89	.17
Pallidum vol.	15.50 (0.97)	17.98 (0.58)	15.70 (0.63)	16.46 (0.48)	2.88	.09	.55	.46
NAcc vol.	5.09 (0.37)	5.22 (0.13)	5.30 (0.38)	5.60 (0.21)	.08	.77	.32	.57

**NB:** the model includes sex and age of the child at MRI assessment, age and race of the pregnant person, prenatal stress class, number of days exposed to the threshold temperature (95°F), socioeconomic status (SES) class, HVI category, and season of conception as covariates. Analyses were based on GEE. Intrafamilial correlations (n = 4 siblings) were adjusted for. Brain volumes are normalized by total intracranial volume. Standard errors for the left and right putamen reflect log10 transformations. NAcc = nucleus accumbens; vol = volume; SE = standard error. Values represent mean volume (%) and standard error*.*

### Interaction effects of Superstorm Sandy (SS) and extreme heat exposure on child brain volume

Extreme heat exposure moderated the association between SS exposure and the volume of the left NAcc (adj-*p* = .05) and left pallidum (adj-*p* = .14) at age 8. When heat exposure was present, SS exposure was associated with a significantly smaller left NAcc and a larger left pallidum. Full numerical results are summarized in Table 4 and visually represented in supplementary figure S3 Fig.

**Table 4:** Interaction Effects of SS and Extreme Heat Exposure on Basal Ganglia Structural Volumes.

Brain region	Extreme Heat	Left hemisphere Mean (SE) (n = 34)		Right hemisphere Mean (SE) (n = 34)		Interaction Statistics					
		Superstorm Sandy Status Exposure Status				Left hemisphere			Right hemisphere		
		Not Exposed	Exposed	Not Exposed	Exposed	Wald X^2^	*p*	adj *p*	Wald X^2^	*p*	adj *p*
Caudate vol.	Unexposed (0)	31.05 (2.02)	34.54 (3.74)	30.19 (1.83)	38.69 (3.01)	.59	.44	.51	2.54	.11	.30
	Exposed (1)	32.68 (0.77)	33.01 (1.95)	33.02 (0.69)	35.75 (2.04)						
Putamen vol.	Unexposed (0)	39.33 (0.02)	51.22 (0.03)	39.76 (0.02)	49.55 (0.03)	2.67	.10	.20	1.73	.19	.25
	Exposed (1)	44.88 (0.01)	50.87 (0.02)	45.89 (0.01)	53.02 (0.02)						
Pallidum vol.	Unexposed (0)	13.83 (1.02)	14.90 (1.05)	14.34 (0.60)	16.29 (0.69)	4.52	.04	**.14**	.05	.83	.83
	Exposed (1)	17.40 (0.46)	21.18 (0.71)	16.19 (0.40)	17.94 (0.72)						
NAcc vol.	Unexposed (0)	5.02 (0.42)	6.15 (0.56)	4.82 (0.50)	6.16 (0.62)	7.47	.006	**.05**	1.79	.18	.29
	Exposed (1)	5.26 (0.17)	4.57 (0.45)	5.53 (0.20)	5.69 (0.68)						

**NB**: Extreme heat was defined as being exposed to at least one day > 95°F during pregnancy. Model includes sex and age of the child at MRI assessment, age and race of the pregnant person, prenatal stress class, objective challenges related to SS, socioeconomic status, number of days exposed to the threshold temperature, HVI category, and season of conception as covariates. Analyses were based on GEE with an adjustment of intrafamilial correlations (n = 4 siblings). Brain volumes are normalized by total intracranial volume. Standard errors for the left and right putamen reflect log10 transformations. FDR corrected p-values (adj *p*) were conducted with the Benjamini-Hochberg procedure with an FDR of 15%. NAcc = nucleus accumbens; vol = volume; SE = standard error. Values represent mean volume (%) and standard error.

## Discussion

There are two major findings. First, SS exposure during pregnancy, but not heat, was associated with larger gray matter volume in the putamen and pallidum bilaterally and the right caudate in middle childhood (approximately age 8) compared to those who were SS unexposed. Second, prenatal exposure to extreme heat amplified the adverse impact SS exposure poses on basal ganglia gray matter volume. Our significant main effects of SS exposure on brain volume are consistent with prior research that demonstrates the influence of prenatal stress exposure on child brain volume. However, this is one of the first studies to demonstrate the effect of prenatal stress in response to a natural disaster on basil ganglia gray matter volumes. Importantly, while we did not find main effects of extreme heat on basal ganglia gray matter volumes, our study was the first to uncover that SS exposure, when coupled with extreme heat, was linked to both discernibly larger (e.g., pallidum) and smaller (e.g., NAcc) basal ganglia gray matter volumes compared to unexposed peers. Our findings provide initial evidence for the importance of evaluating co-occurring risks related to climate change on child brain morphology while also expanding existing knowledge in the field.

### Principle findings

Our finding of significantly larger gray matter volume in various core regions of the basal ganglia (e.g., caudate, putamen, and pallidum) highlights the sensitivity of subcortical structures to climate-related stress experienced during the *in-utero* environment [48,49]. Moreover, our findings extend prior work from both the Project Ice Storm and our SIP study, which found associations between prenatal exposure to the Quebec Ice Storm and Superstorm Sandy and the volume of various subcortical regions, such as the amygdala, hippocampus, and caudate [28,29]. Subcortical structures are well-known to follow an inverted-U-shaped developmental pattern with the basal ganglia reaching peak volumes during adolescence [50–52]. *In-utero* exposure to climate-related stress may disrupt mechanisms of emotion regulation, as suggested in prior literature, impacting brain development, evidenced in our study by larger basal ganglia gray matter volume, compared to unexposed peers, during the latter stages of rapid brain development.

Larger brain volume may promote adaptive functioning or pose a risk to child neurodevelopment. The current perspective remains mixed with findings supporting both outcomes. One prior study found the association between maternal anxiety during pregnancy and child behaviors to be partially mediated by the volume of the left amygdala [27]. Of note, a larger left amygdala volume was related to a reduction in child behavioral difficulties among preschool-aged girls [27], signaling a potential advantage. However, several studies found lager basal ganglia brain volume to instead point to less favorable neurodevelopmental-psychiatric outcomes. Larger right caudate volume, relative to the left caudate, has been associated with higher attentional impulsiveness and risk for ADHD among adults [53]. Moreover, larger putamen volume has been linked with high functioning autism spectrum disorder (ASD) [54]. In addition, greater depressive symptoms have been associated with greater pallidum volume [55]. In line with the literature outlined above, our preliminary findings may suggest larger brain volume in the basal ganglia may reveal emotional and behavioral disruptions and/or serve as a risk factor for impairment. However, we cannot establish this claim with certainty considering larger brain volumes may indicate an adaption to stress. Future work incorporating behavioral assessments is needed to clarify these associations.

Interestingly, unlike SS, extreme heat exposure was not an independent risk factor for altered basal ganglia gray matter volume. As mentioned earlier, the consequences of extreme heat exposure are shown to be sensitive to various environmental, infrastructural, and sociodemographic factors. Despite acknowledging the potential for these confounding effects by adjusting for the New York City HVI level in our relevant models, our non-significant main effect may be due to a myriad of additional unmeasured socioeconomic factors and resources that could have attenuated the association, as access to adequate resources may alleviate the impact of extreme heat. Thus, more comprehensive examination should be pursued in future work. Specifically, stratifying by HVI with a larger and less imbalanced sample may elucidate potential subgroup-specific effects of heat exposure on child brain indices. Moreover, short-term extreme heat exposure, as measured in this study, may be insufficient to cause long-term effects on child brain volume. In addition, the timing of exposure during pregnancy may play an important role. Future work should consider potential delayed effects of prolonged heat exposure (e.g., several days above a threshold temperature) along with trimester-specific risks.

Nonetheless, our interaction models showed that heat exposure amplified the risk SS exposure poses on basal ganglia volume via discernibly larger (i.e., pallidum) and smaller (i.e., NAcc) brain volume. Our finding of reduced gray matter volume in the left NAcc suggests prenatal exposure to SS may reduce the efficiency of this subregion when exposure to extreme ambient heat co-occurs. Reduced efficiency in the NAcc may lead to decreased or inefficient inhibitory projections sent to the pallidum, a primary output region of the NAcc. As a result, it is possible that the pallidum may need to compensate to maintain adequate innervation of motor functioning and motivational, emotional, and reward-related behaviors. The discernibly larger left pallidum gray matter volume seen in the co-exposed group may reflect this potential compensatory innervation [56]. While novel, the findings need to be replicated in independent samples to support these findings. If replicated, the current study will serve as a trailblazer in the field by suggesting initial evidence that co-occurring risks need to be examined together so that their additive or synergistic natures of elevated risks could be formally tested.

## Limitations

The current study has limitations. First, the sample size is small (n = 34), which prevented the exploration of potential sex or trimester-specific risks. Exploring sex-based comparisons is especially important as it plays a crucial role in child developmental processes and morphological characteristics of brain structures. Second, our analysis was limited to structural brain indices. Additional investigation using functional imaging analysis would enhance our understanding by identifying potential subcortical activation patterns in response to prenatal stress. Moreover, considering the interdependent relationship between the brain and behavior, future work should explore whether clinical behavioral indices influence basal ganglia volume and vice-versa. Third, we could not assess brain volume longitudinally, nor could we determine whether the observed brain volume differences exceed typical developmental expectations due to lack of normative comparison data. We are currently conducting a longitudinal follow-up study based on these preliminary findings. Our imaging protocol models the Adolescent Brain Cognitive Development (ABCD) study, a large-scale longitudinal study which tracks the brain development of several thousand 9–10 year olds through adolescence [57]. Our ongoing research will allow us to track brain volume development throughout childhood in relation to these early exposures while also establishing better normative benchmarks for comparison. Fourth, it is important to recognize the difficulty of measuring heat exposure, as there is no standard definition of extreme heat episodes [58]. For example, global studies have associated adverse pregnancy outcomes with exposure to temperatures between 84.2°F (29°C) to 107.6°F (42°C) [59–63]. Despite this, we chose 95°F (35°C) as the threshold temperature because in New York City, specifically, heat-related morbidity (e.g., exhaustion, sun stroke, or heat-related fatigue) is possible at 95°F (35°C) even with lower relative humidity (%) levels [64]. Moreover, the World Health and Meteorological Organizations (WHO; WMO) state the use of electric fans at 95°F (35°C) may worsen heat-related stress [65]. Nonetheless, our findings may not be fully generalizable to populations residing in non-urban environments or regions with different climatic conditions. Fifth, as commented earlier, it is challenging to ascertain the full extent of individual-level heat exposure. Considering not everyone spends the same time indoors and outdoors, has access to inside air conditioning, proper ventilation, and greenspace, it is challenging to precisely discern how long each day, and to what extent (whether indoors or outdoors), the participants were exposed to the threshold temperature. Despite accounting for some of these influences in our current analyses by adjusting for the New York City neighborhood-level HVI, our results should be interpreted with caution. To elucidate this association, future work should consider measuring more precise measures of heat exposure (e.g., outdoor vs indoor occupation and individual-level access to air conditioning, proper ventilation, and cool public spaces) that may influence the degree to which heat exposure impacts child outcomes [19]. Moreover, as mentioned above, potential delayed effects of prolonged heat exposure should be considered since short-term exposures may be insufficient to induce significant changes in brain volumes as evidenced by our study results. Nonetheless, we found a marginally significant main effect between heat exposure and left pallidum volume. However, this finding was not corrected for multiple comparisons, necessitating cautious interpretation. Furthermore, trimester-specific risks should be considered to identify potential critical developmental windows. Additionally, we found a statistically significant age difference between the SS-exposed and non-exposed groups with the SS-exposed group having a lower mean age. Despite adjusting for biological age at MRI assessment in all our analyses, this baseline difference could nonetheless potentially influence the volume of the basal ganglia. Finally, some potential sources of bias remain. This includes selection bias, as participants undergoing neuroimaging may not be fully representative of the general population, measurement error inherent in neuroimaging techniques, and residual confounding from unmeasured factors. However, we believe these biases are more likely to attenuate rather than inflate the observed associations.

## Strengths

This study also has strengths. First, the study’s quasi-experimental design allowed our measures of prenatal stress exposure (i.e., SS and extreme heat) to be objectively defined and naturally assigned in a human population. Second, despite the small sample size, our findings are robust to multiple testing adjustment, intrafamilial correlation (n = 4 siblings), and various pertinent confounders connected to the pregnant individual (e.g., pregnant individual’s age at childbirth, SES, and normative stress class), their offspring (e.g., age at MRI assessment and sex assigned at birth), and their environment (e.g., season of conception and neighborhood heat vulnerability).

## Conclusion

Our findings warrant additional precautions regarding the health dangers of exposure to extreme climate events and natural disasters, specifically for pregnant individuals. Exposure to SS during pregnancy impacted child brain development evidenced by discernibly larger basal ganglia gray matter volume. Extreme heat amplified this risk via increased and decreased volume in various basal ganglia subregions of interest. The world is in a state of climate emergency. Extreme weather events and natural disasters are projected to increase in frequency and magnitude [66]. In addition to promoting initiatives to combat climate change, it is imperative to alert pregnant individuals to the ongoing danger of exposure to extreme climate events in order to protect healthy long-term brain development in young school-aged children. Our results warrant future work to investigate the link between alterations in basal ganglia volume and behavioral disruption, with a potential role of basal ganglia structural variations to either reveal behavioral disruptions or serve as a risk factor for impairment.

## Supporting information

S1 Fig SS Exposure on Basal Ganglia Structural Volumes. Basal ganglia brain volumes are normalized by total intracranial volume. Error bars represent standard errors. Standard errors for the left and right putamen reflect log10 transformations. NAcc = nucleus accumbens; vol = volume. FDR corrected p-values were conducted with the Benjamini Hochberg procedure with an FDR of 15%.
(TIF)

S2 Fig Extreme Heat Exposure on Basal Ganglia Structural Volumes. Basal ganglia brain volumes are normalized by total intracranial volume. Error bars represent standard errors. Standard errors for the left and right putamen reflect log10 transformations. NAcc = nucleus accumbens; vol = volume.
(TIF)

S3 Fig Interaction Effects of SS and Extreme Heat Exposure on Basal Ganglia Structural Volumes. (A) Represents the left and right caudate; (B) left and right putamen; (C) left and right pallidum; (D) left and right nucleus accumbens. Basal ganglia brain volumes are normalized by total intracranial volume. Error bars represent standard errors. Standard errors for the left and right putamen reflect log10 transformations. Vol. = volume. FDR corrected *p*-values were conducted with the Benjamini Hochberg procedure with an FDR of 15%.
(TIF)

S1 Dataset Manuscript Data. (XLSX)
