# Correction: Prenatal exposure to extreme ambient heat may amplify the adverse impact of Superstorm Sandy on basal ganglia volume among school-aged children

**DOI:** 10.1371/journal.pone.0341832

**Published:** 2026-01-27

**Authors:** Donato DeIngeniis, Melissa Blum, Rebecca M. Lee, Ahmed Duke Shereen, Yoko Nomura

In the Funding section, the grant number from the funder NIMH is listed incorrectly. The correct grant number is: R01MH131638.
