# Supplementary figures and images for: Prenatal exposure to extreme ambient heat may amplify the adverse impact of Superstorm Sandy on basal ganglia volume among school-aged children
Source: PLoS One. 2025 Jun 11;20(6):e0324150. doi: 10.1371/journal.pone.0324150 (PMC12157054; doi:10.1371/journal.pone.0324150)

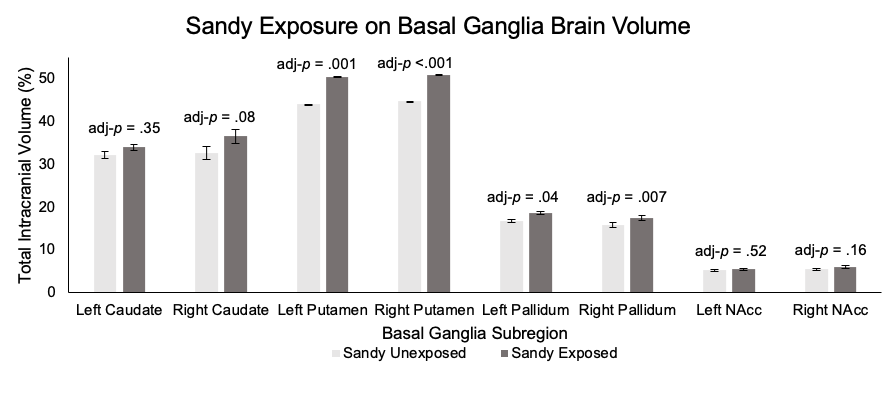

Supplement: S1 Fig — Basal ganglia brain volumes are normalized by total intracranial volume. Error bars represent standard errors. Standard errors for the left and right putamen reflect log10 transformations. NAcc = nucleus accumbens; vol = volume. FDR corrected p-values were conducted with the Benjamini Hochberg procedure with an FDR of 15%. (TIF) [file pone.0324150.s001.tif]

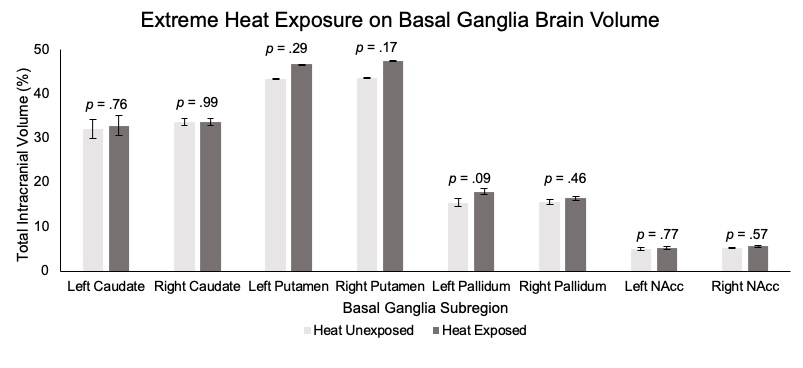

Supplement: S2 Fig — Basal ganglia brain volumes are normalized by total intracranial volume. Error bars represent standard errors. Standard errors for the left and right putamen reflect log10 transformations. NAcc = nucleus accumbens; vol = volume. (TIF) [file pone.0324150.s002.tif]

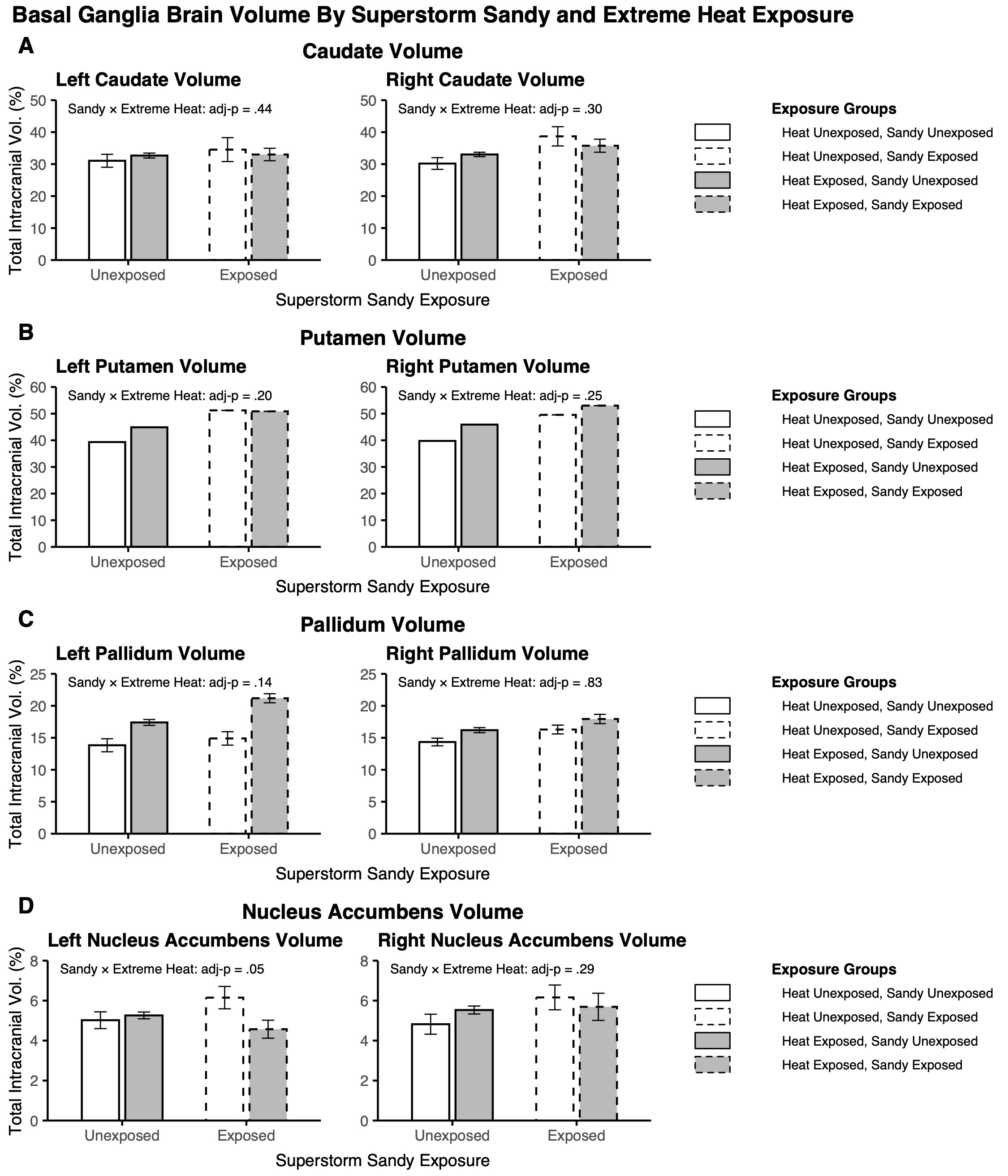

Supplement: S3 Fig — (A) Represents the left and right caudate; (B) left and right putamen; (C) left and right pallidum; (D) left and right nucleus accumbens. Basal ganglia brain volumes are normalized by total intracranial volume. Error bars represent standard errors. Standard errors for the left and right putamen reflect log10 transformations. Vol. = volume. FDR corrected p-values were conducted with the Benjamini Hochberg procedure with an FDR of 15%. (TIF) [file pone.0324150.s003.tif]
